# Diverse Impacts of HIV Latency-Reversing Agents on CD8+ T-Cell Function: Implications for HIV Cure

**DOI:** 10.3389/fimmu.2018.01452

**Published:** 2018-06-22

**Authors:** Genevieve Tyndale Clutton, R. Brad Jones

**Affiliations:** ^1^Department of Microbiology and Immunology, UNC Chapel Hill School of Medicine, Chapel Hill, NC, United States; ^2^Department of Microbiology Immunology and Tropical Medicine, The George Washington University, Washington, DC, United States; ^3^Infectious Disease Division, Weill Cornell Medical College, New York, NY, United States

**Keywords:** T-cells, HIV cure, shock-and-kill, histone deacetylase inhibitor, PKCa

## Abstract

Antiretroviral therapy regimens durably suppress HIV replication, but do not cure infection. This is partially attributable to the persistence of long-lived pools of resting CD4+ T-cells harboring latent replication-competent virus. Substantial clinical and pre-clinical research is currently being directed at purging this viral reservoir by combining pharmacological latency reversal with immune effectors, such as HIV-specific CD8+ T-cells, capable of eliminating reactivated targets—the so-called “shock-and-kill” approach. However, several studies indicate that the latency-reversing agents (LRAs) may affect CD8+ T-cell function. The current review aims to frame recent advances, and ongoing challenges, in implementing “shock-and-kill” strategies from the perspective of effectively harnessing CD8+ T-cells. We review and contextualize findings indicating that LRAs often have unintended impacts on CD8+ T-cell function, both detrimental and beneficial. We identify and attempt to bridge the gap between viral reactivation, as measured by the detection of RNA or protein, and bona fide presentation of viral antigens to CD8+ T-cells. Finally, we highlight factors on the effector (CD8+) and target (CD4+) cell sides that contribute to whether or not infected-cell recognition results in killing/elimination. These perspectives may contribute to an integrated view of “shock-and-kill,” with implications for therapeutic development.

## Introduction

Antiretroviral therapy (ART) has transformed the lives of people living with HIV; however, early hopes that ART might provide a sterilizing cure have proven unfounded (1). A barrier to cure are long-lived populations of latently infected cells harboring integrated provirus, which are not actively producing virions (and, therefore, are impervious to both ART and the immune system) but retain the capacity to do so (2–4). If ART is interrupted, spontaneous activation of these latent cells can lead to viral rebound (5–7).

Recent findings have reignited hopes that a cure for HIV may be possible. Several putative latency-reversing agents (LRAs) have been shown to induce viral RNA production in participants with undetectable viral load on ART (8–10). However, to date most studies have not reported a reduction in the frequency of latently infected cells (as measured by cell-associated replication-competent virus or HIV DNA) following LRA treatment, suggesting that latency reversal alone is unlikely to clear the reservoir. Instead, reactivated cells may need to be actively eliminated by the immune system, a strategy that is known as “shock-and-kill” but may be more precisely characterized as “latency reversal and clearance” (11–13). Supporting this notion, a study using an *in vitro* model of HIV latency demonstrated that latent cells reactivated using Vorinostat did not die from viral cytopathic effects, but could be killed by HIV-specific CD8+ T-cells (14).

CD8+ T-cells can detect and kill virally infected cells with exquisite sensitivity, can be boosted by immunization, and form long-lived “memory” populations capable of rapidly responding to subsequent viral encounters (15, 16). In acute HIV infection, the emergence of HIV-specific CD8+ T-cells coincides with the decline of virus load from peak to set point (17–19), and CD8+ T-cells targeting conserved regions of the HIV proteome (from which the virus is unable to escape without a fitness cost) have been associated with superior virus control in long-term non-progressors (20–25). Furthermore, in a presentation to the 2017 Conference on Retroviruses and Opportunistic Infections, Mothe et al. reported delayed viral rebound following ART interruption in clinical trial participants who received the LRA Romidepsin in combination with a vaccine designed to elicit HIV-specific CD8+ T-cells (26). The vaccine regimen boosted HIV-specific T-cell responses in all participants, and 4 out of 11 were able to maintain viral loads below 2,000 copies/ml for at least 7 weeks after ART interruption, suggesting that the regimen may have impacted the viral reservoir. Thus, HIV-specific CD8+ T-cells are excellent candidates for a HIV cure strategy. However, we and others have reported that some LRAs may have detrimental effects on CD8+ T-cell function, potentially compromising the clearance of reactivated cells. Here, we summarize the current literature, focusing on two leading classes of LRAs: histone deacetylase inhibitors (HDACis) and protein kinase C agonists (PCKa, sometimes also referred to as PKC modulators).

**Figure 1:**
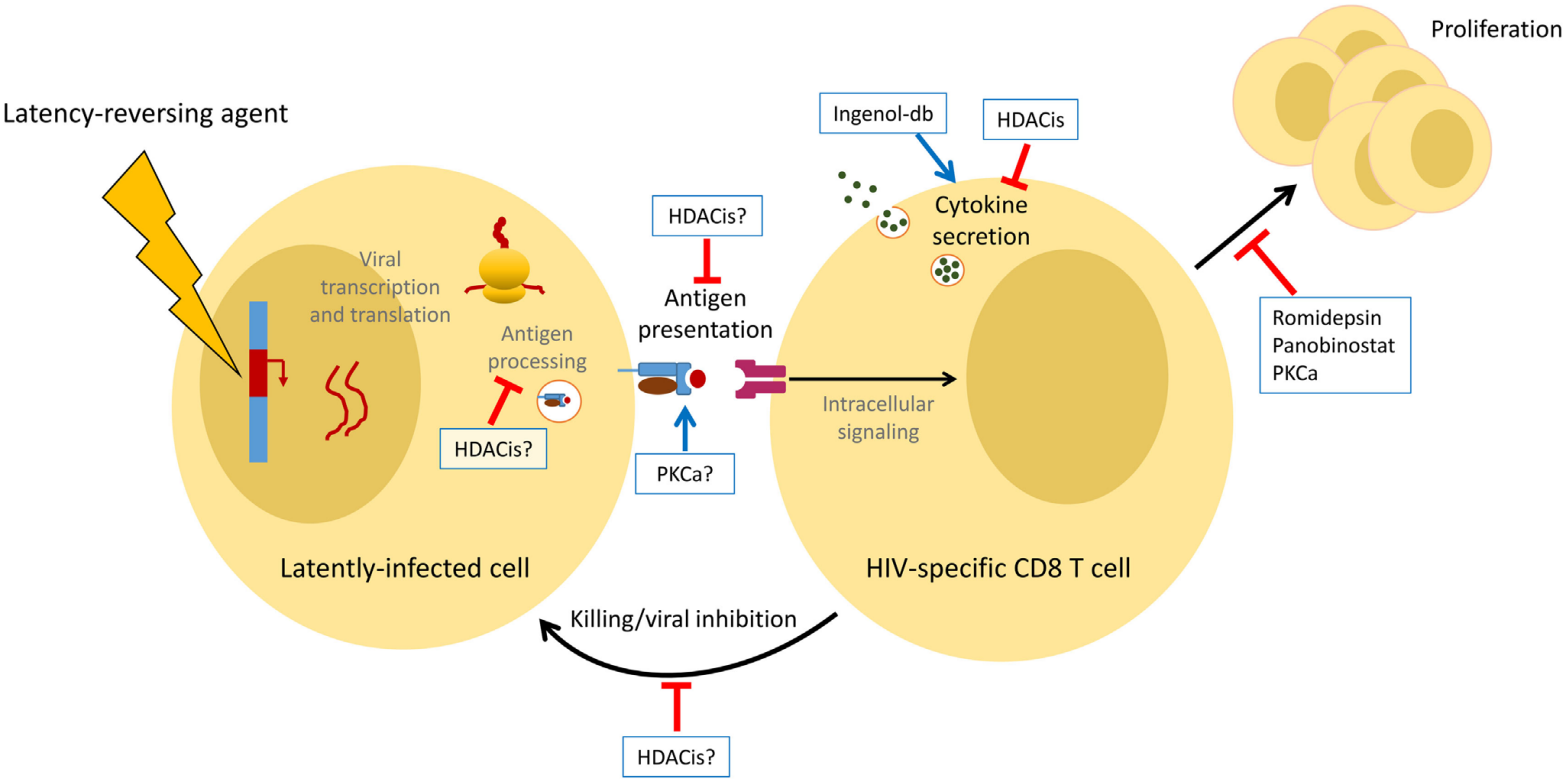
Summary of the effects of latency-reversing agents (LRAs) on antigen-specific CD8+ T-cells *in vitro*. LRAs can either enhance (blue arrows) or inhibit (red bars) multiple facets of the CD8+ T-cell response. Some of these observations have yet to be confirmed by clinical studies.

Histone deacetylase inhibitors block the removal of selected histone acetylation marks, which both allows the recruitment of transcriptional coactivators and inhibits the recruitment of chromosomal silencing complexes (27). Three HDACis (Vorinostat, Romidepsin, and Panobinostat) have been tested as LRAs in clinical trials. PKCa bind to and activate various protein kinase C isoforms, triggering multiple signaling cascades that result in the activation of transcription factors, such as NFκB and ERK1/2 (28). We will discuss three subclasses of PKCa, Bryostatin-1, Prostratin, and Ingenols [primarily Ingenol-B and Ingenol 3,20-dibenzoate (Ingenol-db), two of several Ingenol derivatives proposed as candidate HIV LRAs]. To date, only Bryostatin-1 has been tested as an LRA in clinical trials; the drug failed to enhance PKC activity or increase detection of cell-associated unspliced HIV RNA, indicating that the infusion did not achieve an effective *in vivo* exposure (29). We will summarize both *in vivo* and *in vitro* findings, focusing mostly on studies utilizing primary T-cells and clones, and considering all stages of the T-cell response, from presentation of viral peptides by the infected cell to killing orchestrated by HIV-specific CD8+ T-cells (Figure 1).

## Do LRAs Impair Antigen Presentation?

CD8+ T-cells detect virally infected cells *via* their T-cell receptor (TCR), which recognizes viral peptide (antigen) presented at the infected-cell surface by major histocompatibility class I (MHC-I) molecules (30, 31). Each T-cell population recognizes a specific peptide-MHC combination. For clearance of latently infected cells by CD8+ T-cells to occur, a LRA must induce expression of viral protein that is appropriately presented by MHC-I for a sufficient period of time to be recognized by functional HIV-specific CD8+ T-cells. Notably, HIV virion production is not a prerequisite for viral antigen expression, as resting CD4+ T-cells can transcribe and translate HIV proteins without producing infectious virions, and we and others have previously observed killing of targets infected with replication-defective virus by HIV-specific CD8+ T-cell clones (32–34). The degree to which current latency-reversing regimens induce viral protein production remains uncertain, as the first clinical studies demonstrating latency reversal by HDACis reported increases in viral RNA but did not measure protein (8–10, 35). However, subsequent studies have documented at least some virion release (36).

It is currently unclear whether HDACis such as Vorinostat induce sufficient viral antigen production for recognition of latently infected cells by HIV-specific CD8+ T-cells. Conflicting results have been reported, possibly due to differences in model systems and methods of quantification. For example, using a primary cell latency model, we (Jones and colleagues) recently reported no increase in HIV Gag p24 levels, as measured by ELISA, following Vorinostat treatment (37). However, using a new, highly sensitive digital ELISA method, quantifiable increases in p24 were detected *ex vivo* in resting CD4+ T-cells from ART-suppressed study participants who had received Vorinostat or Panobinostat (36). This suggests that Vorinostat may induce low-level antigen expression below the limit of detection of traditional assays. In contrast, Prostratin treatment induced HIV p24 production at sufficient levels to be measured by standard ELISA (37). Collectively, these studies suggest that LRAs are capable of inducing viral protein expression, but whether this expression occurs at a sufficient level to enable detection by CD8+ T-cells remains inconclusive.

Viral protein expression may not lead to effective antigen presentation. To be detected by HIV-specific CD8+ T-cells, viral antigen must be appropriately processed and presented at the infected-cell surface by MHC-I molecules. The HDACis Panobinostat and Romidepsin transiently reduce MHC-I expression on CD4+ T-cells *in vitro*, whereas PKCa increase expression over several days (38). This may be related to differential activity of peptidases (enzymes that trim peptides prior to loading onto MHC molecules) under these conditions, and thus availability of peptides for MHC-I loading. HDACi treatment of primary CD4+ T-cells results in a 20–60% reduction in peptidase activity compared with untreated cells, whereas this activity is enhanced in cells treated with the PKCa Bryostatin-1 (Boucau et al. NIAID Strategies for an HIV Cure Meeting, 2016). These effects may dampen the abilities of HDACis to induce T-cell recognition of infected cells, while further boosting antigen presentation by PKCa-treated CD4+ T-cells. An alternative approach is to directly quantify antigen presentation by the infected cell. Specific peptide-MHC combinations can be visualized using fluorescently tagged synthetic TCRs (39). Using this method, Yang et al. reported that up to 50 MHC-I complexes presenting the HIV Gag SL9 peptide could be detected on the surface of CD4+ T-cells that had been infected with HIV *in vitro* (40). This method could be utilized to investigate whether antigen is presented at a comparable level on reactivated latently infected cells, whether there are differences in the level of antigen presentation induced by different LRAs, and, crucially, the timing and duration of antigen presentation following LRA administration.

The third consideration when assessing the impact of putative LRAs on HIV antigen expression is whether enough peptide-antigen/MHC complexes are present on the cell surface to facilitate recognition and killing of the infected cell by HIV-specific CD8+ T-cells. Imaging studies of individual cells have shown that three or fewer peptide-MHC complexes can trigger T-cell cytokine production and killing (16, 41, 42), raising the possibility that reactivated infected cells may be recognized by HIV-specific CD8+ T-cells even if expression of the corresponding protein cannot be directly measured by conventional assays. Investigators have begun to address this issue by using CD8+ T-cells as “biosensors” for antigen expression, providing a readout that is directly relevant to “shock-and-kill” strategies. Latently infected CD4+ T-cells are exposed to LRAs, washed, and then co-cultured with CD8+ T-cells to determine whether the cells have been reactivated sufficiently to trigger cytokine production, virus inhibition, or killing. Since HIV-infected cells are typically present at very low frequencies in ART-treated individuals, investigators have usually employed primary cell latency models (where CD4+ T-cells from participants are superinfected and then allowed to recover to a latent state over several days) to assess CD8+ T-cell recognition and infected-cell elimination. Using such a model, Shan et al. demonstrated that antigen-pre-stimulated HIV-specific CD8+ T-cell lines effectively eliminated Vorinostat-reactivated infected cells (14). This finding was supported by an *ex vivo* study showing that Vorinostat sensitized resting CD4+ T-cells from HIV-infected individuals on ART to elimination by autologous CD8+ T-cells (43). In contrast, we (Jones and colleagues) were unable to observe recognition of Vorinostat- or Panobinostat-treated cells by HIV-specific CD8+ T-cell clones, whereas the PKCa Prostratin effectively primed latently infected cells for CD8+ T-cell recognition, as measured by IFN-γ production (37). These conflicting results could be explained by the differences in the latency model, reporter viruses, and HIV-specific CD8+ T-cells (cell lines versus clones) used in the two *in vitro* studies. Interestingly, HDACis induced detectable HIV protein expression in the first *in vitro* study (where Vorinostat-activated cells could be cleared by CD8+ T-cells) but not the second, suggesting that measurable protein expression is a useful indicator of the vulnerability of latently infected cells to clearance *in vitro*.

Even if LRAs induce appropriate antigen production and presentation, clearance could be limited by insufficient frequencies of HIV-specific CD8+ T-cells in HIV-infected individuals on ART [Xu et al., manuscript in preparation (44)]. Additionally, in chronically infected individuals, we and others have observed that HIV-specific CD8+ T-cells exhibit signs of dysfunction that may impair their capacities to eliminate reactivated cells (45–48). To overcome these limitations, researchers have developed bispecific molecules combining a HIV-recognizing component with an element that binds CD3 (part of the TCR complex), so that T-cells of any specificity can be recruited to eliminate latent cells. Using a high-avidity HIV-specific TCR fused with a CD3-specific single chain antibody fragment, Yang et al. reported that resting CD4+ T-cells from HIV-infected individuals on ART pre-treated with Romidepsin plus Bryostatin-1 could be killed by CD8+ T-cells from HIV-seronegative donors (who lack HIV-specific CD8+ T-cells) (40). Similarly, a bispecific antibody targeting HIV gp120 and CD3 facilitated clearance of Vorinostat- or Panobinostat-exposed CD4+ T-cells from HIV+ ART-treated participants by autologous CD8+ T-cells (36). This suggests that these LRA regimens stimulated sufficient antigen presentation for killing in a situation where TCR recognition is optimal and CD8+T-cell numbers are not limiting.

Finally, antigen-presenting cells (and other cells) can modulate antigen-induced T-cell activation by expressing costimulatory or inhibitory molecules (referred to as signal 2) and by producing cytokines (signal 3) (49–51). HDACis and PKCa have been shown to impact signal 2 and signal 3 in cancer and other settings. Vorinostat inhibits expression of the costimulatory molecule CD80 on antigen-presenting cells, though only at supraphysiological doses (52). Vorinostat and Romidepsin can also blunt TLR agonist-induced cytokine production by dendritic cells (52, 53). In contrast, PKCa can enhance costimulatory molecule expression (54), though again, these exposures may not mimic *in vivo* drug pharmacokinetics. We (Clutton and colleagues) observed that Bryostatin-1 also induced a modest but sustained reduction in expression of PD-1, an “immune checkpoint” molecule that attenuates signaling downstream of the TCR, on CD8+ T-cells *in vitro* (38). While HDACis generally induce limited cytokine production *in vitro* when administered at physiological doses, Bryostatin-1 and Ingenol can trigger production of IL-12, which could boost effector CD8+ T-cell responses (38, 55).

Collectively, these observations suggest that some LRAs may induce viral protein production and antigen presentation; however, whether this occurs at a sufficient level to activate HIV-specific CD8+ T-cells has yet to be conclusively established. Adding further complexity, we (Jones and colleagues) have recently reported that while cells harboring defective proviruses can be eliminated, latent cells infected with replication-competent virus appear to be resistant to killing even in the face of powerful LRAs (including mitogens) and potent HIV-specific CD8+ T-cells (34). Potential mechanisms underlying this resistance include HIV Nef-mediated downmodulation of antigen presentation and intrinsic resistance of productively infected cells to killing. In support of the latter hypothesis, Cohn et al. observed that latent cells harboring intact provirus display a distinct, pro-survival transcriptional signature upon reactivation, and Kuo et al. reported that the anti-apoptotic protein BIRC5 can promote survival of HIV-infected CD4+ T-cells (56, 57). More studies are needed to further elucidate the effects of LRAs on antigen processing and presentation and expression of survival-related genes in productively infected latent cells.

## Do LRAs Alter CD8+ T-Cell Function?

Following successful induction of viral transcription, translation, and antigen presentation, the next critical stage of “shock-and-kill” is the recruitment of HIV-specific CD8+ T-cells capable of recognizing viral antigen and responding appropriately by proliferating, secreting antiviral cytokines, and inducing apoptosis of the infected cell. Recent studies have suggested that LRAs may have effects on numerous aspects of CD8+ T-cell function.

### T-Cell Viability

Toxicity is an important consideration when screening LRAs; HDACis such as Vorinostat are used in cancer therapy partly because they can increase expression of pro-apoptotic genes in cancer cells (though not in non-transformed cells) (58). HDACis have been administered to HIV-infected study participants without serious adverse events (8–10, 35, 59, 60), whereas administration of the PKCa Bryostatin has been associated with toxicities including severe myalgia and nausea in patients with persistent or advanced cancers (61, 62). Agents that do not cause clinical toxicity may still have effects on T-cell viability that could compromise the ability to detect and eliminate reactivated HIV-infected cells.

The effects of LRAs on T-cell viability *in vitro* depend on the dose, culture duration, cellular subset, and activation status of the cells. For example, we and others have reported that HDACis are disproportionately toxic to activated T-cells and CD8+ T-cell clones, whereas resting CD4+ T-cells are less susceptible to LRA toxicity (63, 64). Vorinostat is not toxic at doses up to 1 µM (exceeding the maximum plasma concentration reported in clinical studies), whereas we observed that Romidepsin and Panobinostat reduce T-cell and PBMC viability in a dose-dependent manner (37, 38). The effects of PKCa on viability vary between compounds; Ingenols do not appear to be toxic (38, 65), whereas we have observed that Bryostatin-1 and Prostratin induce PBMC and CD8+ T-cell death at higher doses (10 nM or higher for Bryostatin-1 and over 300 nM for Prostratin) (38, 64, 66). Since it is difficult to determine *in vitro* doses that reflect clinically relevant exposures for these compounds, more research is needed to determine whether PKCa would induce significant T-cell death *in vivo*.

### T-Cell Proliferation

Following latency reversal *in vivo*, antigen-specific proliferation of HIV-specific CD8+ T-cells will likely be required to generate sufficient effectors to detect and eliminate the extremely low frequency latently infected cells dispersed in multiple tissues in the body. However, certain LRAs may inhibit T-cell proliferation; indeed HDACis are used to treat various cancers in part because of their anti-proliferative effects (67, 68). We observed that clinically relevant exposures to Romidepsin and Panobinostat can substantially reduce HIV-specific CD8+ T-cell proliferation *in vitro* (38, 63). Vorinostat inhibits proliferation at supraphysiological but not clinically achievable doses (38, 63). The effects of PKCa also differ by drug. Prostratin and Bryostatin-1 induce non-specific CD8+ T-cell proliferation. Since altered PKC signaling has been implicated in leukemic cell growth and differentiation, this observation raises concerns about possible protumorigenic effects of PKCa (69). We (Clutton and colleagues) reported that Prostratin and Bryostatin-1 also limit antigen-specific proliferation, perhaps as a result of toxicity, but more likely by limiting the number of proliferative cycles undergone by antigen-specific CD8+ T-cells. Ingenol-db does not induce non-specific proliferation but also reduces the number of proliferative cycles that undergone by antigen-stimulated CD8+ T-cells (38). Thus, many of the compounds under consideration as LRAs may impair antigen-specific CD8+ T-cell proliferation. The critical questions are whether this occurs *in vivo*, whether the effect would be sufficient to impair clearance, and whether immunization could be used to override these effects by boosting the population of HIV-specific CD8+ T-cells prior to LRA treatment.

### Cytokine Production and Degranulation

Upon encountering viral antigen, HIV-specific CD8+ T-cells secrete cytokines and chemokines to inhibit viral replication, and release granules containing cytotoxic mediators that can induce apoptosis in the infected cell (70, 71). This process is tightly controlled to prevent non-specific effects that could trigger immune pathology [reviewed in Ref. (72)]. We and others have observed that HDACis induce minimal non-specific cytokine production (38, 64). In contrast, the PKCa Prostratin, Ingenol-db, and Bryostatin induce non-specific release of inflammatory cytokines by CD8+ T-cells *ex vivo*, raising the possibility that these agents could trigger immune pathology or increase the susceptibility of bystander CD4+ T-cells to infection by HIV virions released from reactivated cells (38). However, PKCa also reduce expression of the HIV entry co-receptors CD4+ and CCR5, which may render cells less susceptible to infection (38, 65). If tightly monitored and controlled, a modest increase in inflammation might be beneficial in reactivating cells from latency; nevertheless, clinical testing of these agents should be performed with great caution.

The effects of LRAs on antigen-specific CD8+ T-cell function vary even between drugs of the same class. We and others have reported that Vorinostat has no measurable effect on antigen-specific IFN-γ production and degranulation after a pharmacologically relevant exposure *in vitro*, and does not impact *ex vivo* HIV-specific IFN-γ production after multiple *in vivo* doses (35, 38). However, Panobinostat and Romidepsin reduce antigen-specific cytokine production and degranulation *in vitro*, though the effects of Romidepsin appear to be delayed until 10 or more hours after dosing (38, 63). Clinical studies have yet to conclusively support or refute an impact of Romidepsin on HIV-specific CD8+ T-cell responses *in vivo*. In a pilot study of five HIV-infected participants on ART, Romidepsin treatment was associated with a modest reduction in cytokine production by HIV-specific CD8+ T-cells that did not reach statistical significance (10). More recently, Mothe et al. reported a transient decline of 35% in the magnitude of HIV-specific CD8+ T-cell responses following Romidepsin in a therapeutic vaccine plus Romidepsin combination study, though this was a preliminary analysis (26). Collectively, these data suggest that Romidepsin could limit CD8+ T-cell function but that the duration of this impairment may be limited. It will be critical to determine whether viral reactivation and antigen presentation occurs within this timeframe. We (Clutton and colleagues) recently reported that exposure to the PKCa Prostratin and Bryostatin-1 *in vitro* does not affect antigen-specific CD8+ T-cell cytokine production and degranulation, whereas Ingenol-db modestly boosts antigen-specific responses (38). Though this observation will need to be confirmed in further studies, if potential off-target effects can be mitigated, Ingenol derivatives may be attractive candidates capable of both reversing latency and boosting HIV-specific CD8+ T-cell responses against reactivated cells.

### Infected-Cell Elimination

In a study using a primary cell latency model, Shan et al. demonstrated that following reactivation, latently infected CD4+ T-cells do not die by cytopathic effects, but can be killed by HIV-specific CD8+ T-cells (14). In addition to providing a rationale for using T-cell-boosting therapies (such as a vaccine) as part of HIV cure, this study showed that it will be critical to determine whether LRAs have an effect on the killing capacity of CD8+ T-cells. In studies that have addressed this question, it is not always clear whether the effect observed was due to LRAs acting on the infected cell (e.g., affecting antigen processing or presentation) or on HIV-specific CD8+ T-cells directly; however, some have examined effects on CD8+ T-cell killing in isolation. We and others have observed that all three HDACis that have been tested in clinical trials (Vorinostat, Romidepsin, and Panobinostat) can impair killing of infected cells by CD8+ T-cells *in vitro* (37, 63, 66). However, the effect of Vorinostat appeared to be restricted to recently activated T-cells, such as CTL clones, as *in vitro* exposure of primary CD8+ T-cells to the drug did not reduce their viral suppressive capacity, and repeated *in vivo* Vorinostat dosing did not impair the viral suppressive capacity of CD8+ T-cells *ex vivo* (43, 73). Similarly, we (Jones and colleagues) reported that the PKCa Prostratin completely abrogated the viral suppressive capacity of HIV-specific CD8+ T-cell clones, but Blankson and colleagues demonstrated that Prostratin did not reduce suppression by CD8+ T-cells from elite controllers (37, 66, 74). In contrast to Prostratin, Bryostatin-1 impaired the viral suppressive capacity of primary CD8+ T-cells from elite suppressors in a dose-dependent manner, with the addition of Romidepsin further reducing killing (66, 74). Since latently infected cells pre-exposed to Bryostatin-1 plus Romidepsin can be killed by (LRA-unexposed) CD8+ T-cells in the presence of a bispecific fusion molecule [(40); see above], these data suggest that this combination effectively reverses latency but also induces T-cell dysfunction, acting as a “double-edged sword.” In contrast to the other PKCa, the Ingenol derivative Ingenol-B did not reduce the viral suppressive capacity of CD8+ T-cells from elite suppressors (74). Collectively, these observations suggest that T-cell clones, or recently activated CD8+ T-cells, may be more vulnerable to the negative effects of LRAs (in terms of toxicity, proliferative capacity, and viral suppressive capacity) than primary T-cells. The effects of LRAs on CD8+ killing may also depend on the culture conditions *in vitro*, with lower doses and shorter exposures having fewer deleterious effects (37, 66). Once again, it will be vital to determine *in vitro* conditions that reflect clinically relevant exposures.

A direct way of assessing whether combinations of LRAs and T-cells are capable of driving the elimination of cells harboring infectious HIV proviruses from the CD4+ T-cells of HIV-infected individuals on long-term ART is to perform *ex vivo* co-culture experiments, followed by quantitative viral outgrowth assays. We are aware of only three such studies that have measured replication-competent virus (several others having measured viral RNA, which can also be produced by defective proviruses). Two of these studies reported significant reductions in replication-competent virus in a “latency clearance assay” (43, 73), while we (Jones and colleagues) reported an inability to reduce replication-competent virus in a related “HIV eradication assay” (34). There are a number of differences in terms of methodology, LRAs, immune effectors, and study participant populations that could have contributed to these contrasting observations; and work is underway to understand these. Based on a process of elimination of known barriers to infected-cell elimination, our study suggested that cells harboring infectious proviruses may possess some degree of intrinsic-resistance to elimination by T-cells (discussed above). This draws a parallel with the cancer field, where differential susceptibilities of target cells to killing by T-cells is well known as a limiting factor in immunotherapy (75). While further study is needed to evaluate this possibility in the HIV setting, we raise the possibility that in addition to: (i) antigen presentation on infected cells, (ii) functional (cytotoxic) HIV-specific T-cells, and (iii) the intrinsic susceptibility of target cells to cytotoxic effectors (ex. perforin/granzyme or Fas/FasL) may be an important consideration for infected cells to be efficiently eliminated by “shock-and-kill” strategies.

As described above, most LRAs that have been tested to date in viral suppression assays have had detrimental effects. However, we (Jones and colleagues) have observed that the IL-15 superagonist ALT-803 and the TLR-2 ligand Pam_3_CSK_4_ enhance killing of HIV-infected cells by both CD8+ T-cell clones and (in the case of ALT-803) primary CD8+ T-cells from an ART-treated HIV+ participant (37). These agents are exciting candidates that will be examined further in future studies.

## Conclusion

A growing body of work demonstrates that LRAs of the HDACi and PKCa classes can negatively impact CD8+ T-cell function *in vitro*, with more recent clinical studies suggesting that some detrimental effects may also occur *in vivo* (26). Even within the same class, LRAs can vary in their effects; for example, the HDACis Romidepsin and Panobinostat impair antigen-specific CD8+ T-cell function to a greater extent than Vorinostat. However, several outstanding questions must be addressed before we can confidently conclude whether these agents will substantially alter CD8+ T-cell function in a clinical setting. Further studies are needed to assess the impact on T-cells of *in vivo* exposure to LRAs, particularly PKCa, though it is interesting to note a degree of concordance between *in vitro* and *in vivo* studies to date (Table 1). More work is needed to determine *in vitro* conditions that mimic physiological exposures, particularly in tissues other than the blood. This is particularly important for PCKa, which are metabolized so rapidly following infusion that it may be difficult to achieve an effective concentration *in vivo* without unacceptable toxicity (29, 76, 77). Multiple doses of an LRA (or LRAs) will likely be required to substantially deplete the latent reservoir, but few studies to date have examined the effect of repeated exposures on T-cells. The effects of LRAs on HIV-specific CD8+ T-cells must also be balanced with the different efficacies of the various drugs in reversing latency. The current data suggest that, while HDACis may be relatively benign in terms of their effects on CD8+ T-cells, they are less effective latency reversers compared with maximal stimulation (64, 78). PKCa are capable of more robust latency reactivation *in vitro*, but have yet to demonstrate efficacy in a clinical setting (29). Combination therapy with drugs of two or more classes may allow lower doses of the individual agents to be used, preserving their potency as latency reversers while reducing some of their undesirable effects on CD8+ T-cells, though this approach requires further validation (63, 64).

**Table 1:** The effects of histone deacetylase inhibitor (HDACi) and PKCa, administered *in vitro* or *in vivo*, on CD8+ T-cells.

	HDACi		PKCa	
	*In vitro*	*In vivo*	*In vitro*	*In vivo*
Antigen presentation	Reduced MHC class I expression (Romi, Pan) (38)	Unknown	Increased MHC class I expression (38)	Unknown

Costimulatory molecule expression and cytokine production	Reduced (Vor^a^, Romi) (52, 53)	Reduced (Romi, Pan) (53, 79)	Enhanced (Ing, Bryo) (38, 54, 55)	Unknown

T-cell viability	Reduced (Romi, Pan) (63)	Indirect evidence of reduced cell viability in TCL (Vor, Romi) (68)	Reduced (Pro, Bryo) (38, 66)^b^	Unknown

T-cell proliferation	Reduced antigen-specific proliferation (Romi, Pan) (38, 63)	Indirect evidence of reduced cell proliferation in TCL (68, 80)	Induced non-specific proliferation (Pro, Bryo) (38) Reduced antigen-specific proliferation (38)	Unknown

T-cell cytokine production and degranulation	Reduced antigen-specific responses (Romi, Pan) (38, 63)	Modest and transient reduction in HIV-specific cytokine production (Romi) (10, 26) No effect (Vor) (35, 38)	Induced non-specific cytokine production (38) Boosted antigen-specific CD8+ T-cell responses (Ing) (38)	No T-cell-specific data

Infected-cell elimination	Impaired (Romi, Pan) (37, 63, 66) Conflicting reports (Vor) (43, 63, 73)	Unknown^c^	Impaired (Bryo) (66, 74) Conflicting reports (Pro) (37, 66, 74) No effect (Ing) (74)	Unknown

*Unless otherwise stated, the phenotype has been described for all compounds of that class discussed in this review*.

*Vor, vorinostat; Romi, romidepsin; Pan, panobinostat; Pro, prostratin; Ing, ingenols; Bryo, bryostatin-1; TCL, T-cell lymphoma*.

*^a^Doses used possibly higher than clinically achievable exposures*.

*^b^Reference (38) examined total PBMC rather than T-cells specifically*.

*^c^No direct evidence of altered CD8+ T-cell killing capacity as a result of HDACi exposure in vivo*.

A major hurdle to the successful implementation of a “shock-and-kill” strategy is the difficulty of reactivating all cells harboring replication-competent virus without triggering a potentially life-threatening systemic inflammatory response (81, 82). Possible approaches to overcome this issue include reducing systemic effects by targeted delivery of LRAs or other therapeutic drugs to latently infected target cells, or using immunosuppressive drugs such as rapamycin to blunt inflammatory cytokine release without compromising latency reversal (83, 84). However, a more achievable goal may be a so-called “functional cure,” whereby the pool of replication-competent virus is not entirely eradicated but the individual can cease ART permanently without viral rebound or risk of transmission to others (85). In this case, an LRA (or combination of LRAs) would need only to be of sufficient potency to shrink the latent reservoir to the extent that any residual viral replication could be controlled by, for example, potent HIV-specific CD8+ T-cells induced by vaccination or other means (86). It is in this context that the short- and long-term effects of LRAs on CD8+ T-cell function are particularly pertinent.

One intriguing finding of the studies to date is that the timing and duration of effects on antigen-specific CD8+ T-cells varies even between LRAs of the same class (38, 63). Whether this impairment coincides with the period during which antigen is presented by reactivated cells could be the major determinant of whether HIV-specific CD8+ T-cells are able to eliminate latent cells. Of note, Sung et al. recently reported that CD8+ T-cells from HIV-infected study participants on ART could suppress viral replication in autologous CD4+ T-cells treated with Vorinostat within 24 h, suggesting that both antigen presentation and CD8+ T-cell killing can occur within this timeframe (43). It is hoped that additional studies examining the kinetics of antigen presentation (or “window of vulnerability”) following LRA treatment will continue to shed light on this issue.

The ultimate question with regards to detrimental effects of LRAs on CD8+ T-cells is whether they will be of sufficient magnitude to critically compromise “shock-and-kill” HIV cure efforts. As ongoing research has furthered our knowledge of the latent reservoir, it has become increasingly clear that a successful “shock-and-kill” strategy will likely require a multi-pronged approach including not only latency reversal but also strategies such as immunization and/or “immune checkpoint” blockade to boost HIV-specific CD8+ T-cell responses. It will be important to examine the effects of these combinatorial strategies on CD8+ T-cells. For example, “immune checkpoint” inhibitors block inhibitory signals from proteins such as PD-1, and have been demonstrated to enhance HIV-specific CD8+ T-cell function (45, 87). These inhibitors could negate the detrimental effects of some LRAs on antigen-specific T-cell proliferation and killing but could also potentially exacerbate the non-specific T-cell responses induced by agents such as Bryostatin-1, therefore, increasing the risk of serious adverse events (38). More promisingly, a recent clinical trial combining Romidepsin with a HIV vaccine showed that although the frequency of vaccine-specific CD8+ T-cells transiently declined after Romidepsin infusion, these responses were efficiently boosted by a second immunization. Furthermore, one-third of the participants experienced delayed viral rebound following ART interruption (26). Though these results were preliminary, and further studies are needed to confirm the findings, this trial suggested that, when fully optimized, a protocol combining LRAs and HIV-specific CD8+ T-cells could provide durable viral suppression *in vivo*. Finally, it may be necessary to harness additional immune effectors such as NK cells and γδ T-cells to achieve optimal clearance of reactivated latent cells (88, 89). To date, there have been fewer studies of the effects of LRAs on these cell populations, though currently available data suggest that HDACis may have detrimental effects on NK cell function (52, 53, 90).

The search for a cure for HIV has been underway since the start of the epidemic, with numerous hurdles and disappointments as well as promising breakthroughs (91). However, the discovery of compounds that can reverse latency has re-energized the field. If latency reversal can be combined with effective, durable HIV-specific CD8+ T cell responses, this elusive goal may finally be attainable.

## Author Contributions

All authors listed have made a substantial, direct, and intellectual contribution to the work and approved it for publication.

## Conflict of Interest Statement

The authors declare that the research was conducted in the absence of any commercial or financial relationships that could be construed as a potential conflict of interest.
